# Effectiveness and Safety of Oral Azvudine for Elderly Hospitalized Patients With COVID‐19: A Multicenter, Retrospective, Real‐World Study

**DOI:** 10.1002/advs.202404450

**Published:** 2025-02-11

**Authors:** Ranran Sun, Haiyu Wang, Junyi Sun, Mengzhao Yang, Shixi Zhang, Xinjun Hu, Bo Yu, Zhan Song, Na Han, Hong Luo, Ming Cheng, Guangming Li, Guotao Li, Yiqiang Yuan, Lili Liang, Yanyang Zhang, Donghua Zhang, Silin Li, Quancheng Kan, Hongxia Liang, Zhigang Ren

**Affiliations:** ^1^ Department of Infectious Diseases State Key Laboratory of Antiviral Drugs Pingyuan Laboratory The First Affiliated Hospital of Zhengzhou University Zhengzhou 450052 China; ^2^ Department of Infectious Diseases Shangqiu Municipal Hospital Shangqiu 476000 China; ^3^ Department of Infectious Diseases The First Affiliated Hospital College of Clinical Medicine Henan University of Science and Technology Luoyang 471003 China; ^4^ Department of Pharmacy The First Affiliated Hospital of Zhengzhou University Zhengzhou 450052 China; ^5^ Department of Gastrointestinal Surgery Nanyang Central Hospital Nanyang 473009 China; ^6^ Guangshan County People's Hospital Guangshan County Xinyang 465450 China; ^7^ Department of Medical Information The First Affiliated Hospital of Zhengzhou University Zhengzhou 450052 China; ^8^ Department of Liver Disease The Affiliated Infectious Disease Hospital of Zhengzhou University Zhengzhou 450052 China; ^9^ Department of Infectious Diseases Luoyang Central Hospital Affiliated of Zhengzhou University Luoyang 471000 China; ^10^ Department of Cardiovascular Medicine Henan Provincial Chest Hospital Affiliated of Zhengzhou University Zhengzhou 450008 China; ^11^ Henan Center for Disease Control and Prevention Zhengzhou 450016 China; ^12^ Department of Infectious Diseases Anyang City Fifth People's Hospital Anyang 455000 China; ^13^ Department of Respiratory and Critical Care Medicine Fengqiu County People's Hospital Xinxiang 453300 China; ^14^ Henan Key Laboratory of Precision Clinical Pharmacy Zhengzhou University Zhengzhou 450052 China

**Keywords:** Azvudine, COVID‐19, effectiveness, elderly, real‐world, safety

## Abstract

Azvudine is recommended as a priority treatment for patients with Coronavirus Disease 2019 (COVID‐19) during Omicron wave in China, but its efficacy and safety in elderly patients is unknown. In this multicenter, retrospective study, we identified 19763 elderly patients (aged over 60 years) with COVID‐19 from nine hospitals in Henan Province, China. The primary outcome is all‐cause death and the secondary outcome is composite disease progression. After propensity score matching, 4109 Azvudine recipients and 4109 matched controls is included, with average age of 75.15 years. Kaplan–Meier analysis reveales a notably survival and progression‐free benefit in Azvudine treatment. The Cox analysis shows that compared with controls, Azvudine recipients have a 33% lower risk of all‐cause death (95% confidence Interval (CI): 0.580–0.772, p < 0.001), but have no significant difference in composite disease progression (hazard ratio: 0.93, 95% CI: 0.833‐1.046, p = 0.234). Subgroup analysis suggested Azvudine have a stronger protective effect in patients concomitant with antibiotics. Three sensitive analyses confirm the robustness of the findings. The safety of Azvudine in elderly patients is acceptable. These findings indicate that Azvudine therapy can reduce the rate of all‐cause death in hospitalized elderly patients with COVID‐19, and without obvious adverse events.

## Graphical abstract

This multicenter, retrospective, real‐world study enrolls the largest hospitalized elderly cohort of COVID‐19 patients from nine hospitals in Henan Province to investigate the effectiveness and safety of Azvudine. The results show that compared controls, Azvudine treatment can reduce the rate of all‐cause death in elderly patients with COVID‐19, and without obvious adverse events.

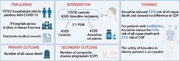

## 1 Introduction

The global Coronavirus Disease 2019 (COVID‐19) pandemic, caused by severe acute respiratory syndrome coronavirus 2 (SARS‐CoV‐2), has lasted over four years and lead to more than 774 million infections and 7 million deaths.^[^ ^1^ ^]^ Although the World Health Organization (WHO) declared in May 2023 that the COVID‐19 epidemic no longer constitutes a public health emergency of international concern, the epidemic dominated by the Omicron strain continues to endanger people's lives and health, especially the elderly.^[^ ^2^ ^]^ In the fifth wave of the COVID‐19 epidemic caused by the Omicron strain in Hong Kong, China, more than 95% of deaths occurred in people aged 60 years or older.^[^ ^3^ ^]^ The elderly usually have underlying diseases and lower immunity, and are at greater risk of severe illness and death after being infected with SARS‐CoV‐2.^[^ ^4^ ^]^

In December 2022, mainland China experienced the first large wave of COVID‐19 infections caused by variants BF.7 and BA.5.2. Azvudine and Nirmatrelvir/ritonavir (Paxlovid) are the main antiviral drugs used in China at this time.^[^ ^5^ ^]^ Paxlovid, a 3‐chymotrypsin–like cysteine protease enzyme inhibitor, have been shown the effectiveness in treatment of COVID‐19 patients in both clinical trials and real‐world studies.^[^ ^6^ ^]^ Unfortunately, Paxlovid is expensive and in short supply, making it inaccessible to patients in developing countries. Therefore, it is imperative to develop alternative drugs that can effectively treat COVID‐19. Azvudine, as the first independently developed oral small molecule anti‐COVID‐19 drug in China, received conditional approval in July 2022 for the treatment of adult patients with common COVID‐19 in China. Multiple clinical trials and real‐world studies have found that Azvudine can effectively reduce the time to nucleic acid negative conversion, all‐cause mortality, and composite disease progression.^[^ ^7^ ^]^ However, there are limited data on the effectiveness and safety of Azvudine in people over 60 years of age.

In this study, we aimed to evaluate the clinical effectiveness and safety of Azvudine in elderly hospitalized patients with COVID‐19 in a large‐scale, muti‐center, retrospective cohort in Henan, China. This is the real‐world experience with Azvudine in the treatment of elderly COVID‐19 patients during the first wave of Omicron in China in 2022.

## 2 Results

### 2.1 Baseline Characteristics of The Overall Cohort

**Figure 1:**
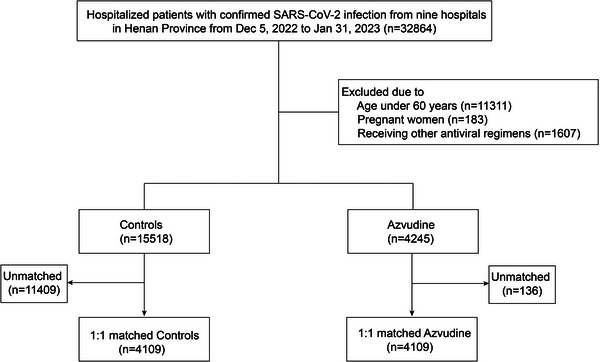
The flowchart of study design.

From December 5, 2022 and January 31, 2023, we totally collected 32 864 confirmed COVID‐19 patients admitted to nine Hospitals in Henan Province, and enrolled 15 518 patients receiving standard treatment and 4245 patients receiving standard treatment plus Azvudine according to inclusion and exclusion criteria (**Figure** **1**). To control for the effects of confounders, we performed propensity score matching (PSM) analysis and finally included 4109 patients in the control group and 4109 patients in the Azvudine group.

We compared the baseline characteristic at admission of two groups and the results were showed in the **Table** **1**. Before matching, most baseline characteristics of the two groups were unbalanced, including age, gender, body mass index (BMI), severity, vaccination doses, concomitant systemic steroid, concomitant antibiotics, comorbidities other than autoimmune diseases, neutrophil, glucose, creatine, glomerular filtration rate, C‐reactive protein, procalcitonin, prothrombin time, activated partial thromboplastin time, cholesterol, alkaline phosphatase, albumin, total bilirubin. After matching, the baseline characteristics of the two groups of patients reached a balance with p > 0.05, standardized mean differences (SMD) < 10% (Figure S1, Supporting Information). The mean age of patients was 75.06 years in control group and 75.24 years in Azvudine group.

**Table 1:** Baseline characteristics of elderly patients with COVID‐19 before and after propensity score matching.

Characteristics	Before matching			After 1:1 matching		
	Control (n = 15 518)	Azvudine (n = 4245)	P value	Control (n = 4109)	Azvudine (n = 4109)	P value
**Age, mean (standard deviation, SD), year**	73.92 (8.72)	75.29 (8.55)	**<0.001**	75.06 (8.85)	75.24 (8.55)	**0.369**
**Gender, n (%)**			**<0.001**			**0.838**
**Male**	8913 (57.4)	2614 (61.6)		2526 (61.5)	2516 (61.2)	
**Female**	6605 (42.6)	1631 (38.4)		1583 (38.5)	1593 (38.8)	
**BMI, mean (SD), kg m^−2^**	23.37 (3.83)	23.64 (3.84)	**<0.001**	23.68 (3.85)	23.63 (3.83)	**0.547**
**Severity at admission, n (%)**			**<0.001**			**0.82**
**Mild**	1319 (8.5)	206 (4.9)		193 (4.7)	204 (5.0)	
**Moderate**	11 721 (75.5)	2901 (68.3)		2831 (68.9)	2834 (69.0)	
**Severe^a^**	2478 (16.0)	1138 (26.8)		1085 (26.4)	1071 (26.1)	
**Vaccination doses, n (%)**			**<0.001**			**0.967**
**0 dose**	4102 (26.4)	1282 (30.2)		1267 (30.8)	1234 (30.0)	
**1 dose**	953 (6.1)	288 (6.8)		267 (6.5)	277 (6.7)	
**2 doses**	2221 (14.3)	542 (12.8)		530 (12.9)	532 (12.9)	
**3 doses**	8043 (51.8)	2081 (49.0)		1992 (48.5)	2017 (49.1)	
**4 doses**	196 (1.3)	51 (1.2)		52 (1.3)	48 (1.2)	
**5 doses**	3 (0.0)	1 (0.0)		1 (0.0)	1 (0.0)	
**Concomitant systemic steroid, n (%)**			**<0.001**			**0.246**
**No**	11 820 (76.2)	2326 (54.8)		2376 (57.8)	2323 (56.5)	
**Yes**	3698 (23.8)	1919 (45.2)		1733 (42.2)	1786 (43.5)	
**Concomitant antibiotics, n (%)**			**<0.001**			**0.477**
**No**	9200 (59.3)	1848 (43.5)		1788 (43.5)	1821 (44.3)	
**Yes**	6318 (40.7)	2397 (56.5)		2321 (56.5)	2288 (55.7)	
**Comorbidities, n (%)**						
**Diabetes**	3663 (23.6)	1151 (27.1)	**<0.001**	1130 (27.5)	1124 (27.4)	**0.902**
**Hypertension**	6360 (41.0)	2015 (47.5)	**<0.001**	1876 (45.7)	1924 (46.8)	**0.298**
**Liver diseases**	1600 (10.3)	358 (8.4)	**<0.001**	385 (9.4)	358 (8.7)	**0.317**
**Cardio‐cerebral diseases**	6937 (44.7)	1526 (35.9)	**<0.001**	1507 (36.7)	1524 (37.1)	**0.715**
**Kidney diseases**	1953 (12.6)	1174 (27.7)	**<0.001**	999 (24.3)	1053 (25.6)	**0.177**
**Primary malignant tumor**	3415 (22.0)	369 (8.7)	**<0.001**	387 (9.4)	369 (9.0)	**0.516**
**Chronic respiratory diseases**	2876 (18.5)	895 (21.1)	**<0.001**	880 (21.4)	854 (20.8)	**0.499**
**Autoimmune diseases**	440 (2.8)	107 (2.5)	**0.291**	104 (2.5)	99 (2.4)	**0.776**
**Laboratory parameters, mean (SD)**						
**Neutrophil, ×10^9^/L**	5.67 (5.16)	5.91 (4.05)	**0.006**	5.93 (4.17)	5.91 (4.06)	**0.806**
**Lymphocyte, ×10^9^/L**	1.52 (15.47)	1.34 (16.57)	**0.512**	1.16 (1.58)	1.36 (16.84)	**0.449**
**Glucose, mmol L^−1^**	7.32 (3.89)	8.01 (4.13)	**<0.001**	8.01 (4.54)	7.95 (4.10)	**0.543**
**High‐density lipoprotein, mmol L^−1^**	1.25 (2.68)	1.18 (2.08)	**0.122**	1.17 (2.02)	1.18 (2.11)	**0.762**
**Low‐density lipoprotein, mmol L^−1^**	2.43 (2.50)	2.42 (2.75)	**0.81**	2.44 (2.69)	2.42 (2.79)	**0.736**
**Alanine aminotransferase, IU/L**	33.55 (85.23)	34.44 (66.23)	**0.532**	34.61 (82.71)	34.44 (67.04)	**0.922**
**Aspartate aminotransferase, IU/L**	42.37 (122.06)	40.37 (87.11)	**0.317**	40.61 (88.35)	40.32 (88.45)	**0.879**
**Creatine, µmol L^−1^**	99.72 (194.99)	92.40 (118.86)	**0.02**	91.28 (95.47)	92.90 (120.64)	**0.499**
**Glomerular filtration rate, ml min^−1^**	105.54 (144.45)	97.34 (121.73)	**0.001**	95.64 (116.84)	97.63 (122.94)	**0.451**
**C‐reactive protein, mg L^−1^**	49.49 (62.00)	53.56 (64.38)	**<0.001**	54.19 (65.61)	53.52 (64.70)	**0.641**
**Procalcitonin, ng ml^−1^**	1.90 (10.48)	1.20 (6.93)	**<0.001**	1.17 (6.62)	1.22 (7.03)	**0.74**
**Prothrombin time, s**	14.67 (8.14)	17.58 (10.90)	**<0.001**	16.76 (10.61)	17.12 (10.63)	**0.126**
**Activated partial thromboplastin time, s**	29.31 (10.98)	26.21 (11.79)	**<0.001**	27.11 (11.04)	26.62 (11.73)	**0.052**
**Cholesterol, mmol L^−1^**	4.37 (4.48)	4.11 (3.08)	**<0.001**	4.14 (2.46)	4.12 (3.12)	**0.75**
**Triglyceride, mmol L^−1^**	1.72 (4.58)	1.63 (5.12)	**0.302**	1.61 (3.53)	1.65 (5.20)	**0.648**
**Alkaline phosphatase, IU/L**	91.10 (72.94)	82.53 (59.88)	**<0.001**	85.10 (56.11)	82.94 (60.57)	**0.094**
**Gamma‐glutamyl transpeptidase, IU/L**	52.27 (91.45)	54.19 (85.68)	**0.22**	55.75 (103.80)	53.78 (84.26)	**0.344**
**Albumin, g L^−1^**	38.36 (20.68)	37.13 (31.94)	**0.003**	37.34 (21.07)	37.29 (32.43)	**0.928**
**Total bilirubin, µmol L^−1^**	14.42 (26.12)	12.64 (14.33)	**<0.001**	12.81 (15.00)	12.64 (14.49)	**0.618**

^a)^ Patients with severe disease in this study are defined as patients with severe and critical disease in the guidelines.

### 2.2 All‐Cause Death and Composite Disease Progression

**Figure 2:**
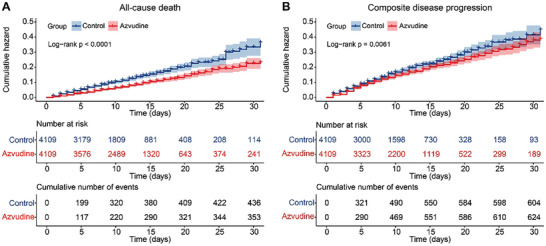
Kaplan–Meier curves of control and Azvudine groups. Cumulative hazard of all‐cause death A) and composite disease progression B).

**Figure 3:**
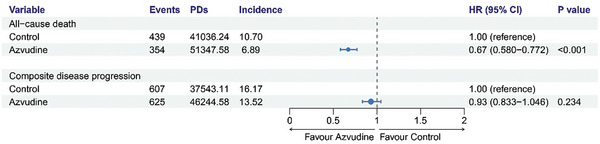
Multivariate Cox proportional hazards regression analysis of all‐cause death and composite disease progression in control and Azvudine groups. Adjusted for all baseline covariates in Table 1. A) Hazard Ratio of all‐cause death. B) Hazard Ratio of composite disease progression. HR: Hazard Ratio; 95%CI: 95% confidence interval. PDs: Person‐days. Incidence: events/per 1000 PDs.

At the end of the follow‐up, 439 patients died in control group and 354 patients died in Azvudine group. We used the Kaplan‐Meier method and log‐rank test to compare outcome events between the two groups. All‐cause mortality was significantly lower in the Azvudine group than in the control group (log‐rank p < 0.0001) (**Figure** **2**A). The crude incidence rate of all‐cause death outcome was 10.70 per 1000 person‐days in control group versus 6.89 per 1000 person‐days in the Azvudine group (**Figure** **3**). We further evaluated the hazard ratio of two treatment on all‐cause mortality by using multivariate Cox regression models with the adjustment for all baseline variables. The result showed the risk of death was 0.67 (95% confidence interval (CI): 0.580‐0.772) in Azvudine group compared with control group (p < 0.001) (Figure 3).

A total of 607 events of composite disease progression was occurred in control group and 625 events in Azvudine group. The risk of the composite disease progression outcome was significantly lower in Azvudine recipients than with nonrecipients by Kaplan‐Meier method (p = 0.0061) (Figure 2B). The crude incidence rate of composite disease progression outcome was 16.17 per 1000 person‐days in control group versus 13.52 per 1000 person‐days in the Azvudine group. However, in the multi‐adjusted Cox proportional hazard model, there was no significant difference on composite disease progression outcome in Azvudine group versus control group (hazard ratio (HR): 0.93, 95% CI: 0.833‐1.046, p = 0.234) (Figure 3).

### 2.3 Subgroup Analysis

In subgroup analysis, patients were grouped by gender, severity, vaccination doses, concomitant systemic steroid, concomitant antibiotics, and comorbidities (**Table** **2**). The results showed compared with patients without antibiotics, a stronger therapeutic benefit of Azvudine treatment for all‐cause death (p for interaction = 0.015) and composite disease progression (p for interaction = 0.015) was observed in the patients with antibiotics. For patients with antibiotics, the risk of all‐cause death reduced 43% in Azvudine group compared with control group (HR: 0.57, 95% CI: 0.48‐0.68). Moreover, Azvudine treatment was associated with a 21% lower risk of composite disease progression compared with control group (HR: 0.79, 95% CI:0.68–0.90).

**Table 2:** Subgroup analyses for Azvudine treatment effect on all‐cause death and composite outcomes in the elderly COVID‐19 patients according to baseline characteristics.

Characteristic	All‐Cause death		Composite disease progression	
	HR (95%CI) ^a)^	P value for interaction	HR (95%CI) ^a)^	**P value for interaction**
**Gender**				
**Male**	0.68 (0.58−0.81)	**0.196**	0.90 (0.79−1.03)	**0.05**
**Female**	0.55 (0.43−0.71)		0.76 (0.62−0.93)	
**Severity at admission**				
**Mild**	0.94 (0.41−2.15)	**0.219**	0.67 (0.28−2.4)	**0.649**
**Moderate**	0.78 (0.59−1.03)		0.95 (0.72−1.25)	
**Severity**	0.61 (0.52−0.73)		0.90 (0.80−1.02)	
**Vaccination doses**				
**None**	0.62 (0.50−0.78)	**0.744**	0.93 (0.78−1.12)	**0.934**
**One dose**	0.60 (0.37−0.96)		0.80 (0.53−1.21)	
**Two doses**	0.51 (0.34−0.77)		0.83 (0.60−1.14)	
**Three doses**	0.72 (0.57−0.90)		0.82 (0.69−0.98)	
**Four doses**	0.24 (0.02−2.35)		0.66 (0.17−2.48)	
**Concomitant antibiotics, n (%)**				
**No**	0.83 (0.65−1.06)	**0.015**	1.03 (0.85−1.25)	**0.015**
**Yes**	0.57 (0.48−0.68)		0.79 (0.68−0.90)	
**Concomitant systemic steroid, n (%)**				
**No**	0.71 (0.58−0.87)	**0.096**	0.89 (0.76−1.04)	**0.301**
			
**Yes**	0.57 (0.47−0.70)	0.82 (0.69−0.96)	
**Diabetes, n (%)**				
**No**	0.61 (0.52−0.72)	**0.421**	0.81 (0.71−0.92)	**0.1**
**Yes**	0.71 (0.75−0.91)		0.99 (0.80−1.22)	
**Hypertension, n (%)**				
**No**	0.64 (0.53−0.77)	**0.95**	0.86 (0.74−1.11)	**0.846**
**Yes**	0.64 (0.52−0.79)		0.86 (0.72−1.01)	
**Liver diseases, n (%)**				
**No**	0.64 (0.55−0.75)	**0.967**	0.85 (0.76−0.96)	**0.85**
**Yes**	0.65 (0.47−0.90)		0.89 (0.66−1.21)	
**Cardio‐cerebral diseases, n (%)**				
**No**	0.63 (0.52−0.78)	**0.97**	0.86 (0.74−1.11)	**0.725**
**Yes**	0.63 (0.51−0.76)		0.84 (0.71−0.99)	
**Kidney diseases, n (%)**				
**No**	0.67 (0.57−0.79)	**0.287**	0.91 (0.79−1.04)	**0.171**
**Yes**	0.57 (0.44−0.73)		0.75 (0.62−0.91)	
**Primary malignant tumor, n (%)**				
**No**	0.62 (0.54−0.72)	**0.229**	0.84 (0.75−0.94)	**0.198**
**Yes**	0.82 (0.49−1.35)		1.06 (0.70−1.62)	
**Chronic respiratory diseases, n (%)**				
**No**	0.64 (0.55−0.75)	**0.774**	0.84 (0.74−0.95)	**0.635**
**Yes**	0.60 (0.42−0.85)		0.92 (0.72−1.17)	
**Autoimmune diseases, n (%)**				
**No**	0.63 (0.55−0.73)	**0.351**	0.85 (0.76−0.96)	**0.969**
**Yes**	1.11 (0.37−3.31)		0.85 (0.37−1.93)	

^a^ ^)^Efficacy outcome for Azvudine versus controls in different subgroup. Reference group: controls.

### 2.4 Sensitivity Analysis

We performed three sensitivity analyses, all of which verified the robustness of our results. First, we used the mean to impute missing data (Table S1, Supporting Information), and Kaplan‐Meier results showed that the Azvudine group was associated with lower all‐cause death (log‐rank p < 0.0001, Figure S2A, Supporting Information) and composite disease progression (log‐rank p = 0.0021, Figure S2B, Supporting Information) compared with control group. Multivariate Cox regression models showed that after adjusting for all baseline variables, Azvudine was associated with a lower risk of all‐cause death compared with the control group (HR: 0.69, 95% CI: 0.594‐0.791, p < 0.001) (Figure S3, Supporting Information), but did not significantly reduce the risk of disease progression consistent with the control group (HR: 0.95, 95% CI: 0.845‐1.062, p = 0.354) (Figure S3, Supporting Information).

Second, we used a Probit regression model for propensity matching (Table S2, Supporting Information). Both the Kaplan‐Meier curve (log‐rank p < 0.0001, Figure S4A, Supporting Information) and Cox regression (HR: 0.70, 95% CI: 0.603‐0.803, p < 0.001, Figure S5, Supporting Information) showed that Azvudine had a lower risk of all‐cause death. The risk of composite disease progression was obviously different in Azvudine treatment versus controls by Kaplan‐Meier curve analysis (log‐rank p = 0.0081, Figure S4B, Supporting Information), but was not significant different by Cox regression analysis (HR: 0.96, 95% CI: 0.856‐1.074, p = 0.468, Figure S5, Supporting Information).

Third, we excluded patients who were discharged from the hospital or died after the same day of medication (Table S3, Supporting Information). Compared with control group, Azvudine was associated with a lower risk of all‐cause death (log‐rank p < 0.0001, Figure S6A (Supporting Information); HR: 0.62, 95% CI: 0.533‐0.712, p < 0.001, Figure S7, Supporting Information) and composite disease progression (log‐rank p = 0.00021, Figure S6B (Supporting Information); HR: 0.87, 95% CI: 0.773‐0.972, p = 0.015, Figure S7, Supporting Information).

### 2.5 Safety Analysis

To assess the safety of Azvudine in a real‐world population, we collected adverse event rates in both groups (**Table** **3**). The results of total adverse events showed that compared with control group, Azvudine group had lower incidences of hypophosphatemia (p = 0.005), had higher incidences of lymphocyte count decreased (p < 0.001), lymphocyte count increased (p = 0.013), anemia (p = 0.009), hypokalemia (p < 0.001), ALT increased (p = 0.006), and hypertriglyceridemia (p = 0.011). As for adverse events graded ≥ 3, Azvudine group had a higher risk of lymphocyte count decreased (p < 0.001) and hypokalemia (p < 0.001) versus control.

**Table 3:** Incidence of adverse event of elderly COVID‐19 patients in control and Azvudine groups.

Adverse events (n, %)	Available data^a)^		All grades			Grade ≥ 3^b)^		
	Control	Azvudine	Control	Azvudine	P value	Control	Azvudine	P value
Lymphocyte count decreased	3603	3770	861 (24%)	1089 (29%)	<0.001	419 (12%)	610 (16%)	<0.001
Lymphocyte count increased	3603	3770	40 (1.1%)	68 (1.8%)	0.013	3 (<0.1%)	2 (<0.1%)	0.7
Neutrophil count increased	1118	1544	56 (5.0%)	60 (3.9%)	0.2	11 (1.0%)	9 (0.6%)	0.2
PLT count decreased	1573	2192	164 (10%)	207 (9.4%)	0.3	0 (0%)	0 (0%)	0 (0%)
Anemia	1336	1589	491 (37%)	659 (41%)	0.009	108 (8.1%)	134 (8.4%)	0.7
Hypophosphatemia	494	646	120 (24%)	113 (17%)	0.005	0 (0%)	0 (0%)	
Hypokalemia	2172	2543	406 (19%)	599 (24%)	<0.001	156 (7.2%)	256 (10%)	<0.001
Hyperkalemia	2172	2543	93 (4.3%)	105 (4.1%)	0.8	30 (1.4%)	30 (1.2%)	0.5
ALT increased	1360	1986	243 (18%)	432 (22%)	0.006	17 (1.3%)	30 (1.5%)	0.5
AST increased	1454	2100	240 (17%)	348 (17%)	>0.9	28 (1.9%)	37 (1.8%)	0.7
ALP increased	1340	1914	63 (4.7%)	114 (6.0%)	0.12	0 (0%)	0 (0%)	
GGT increased	1092	894	129 (12%)	180 (13%)	0.4	10 (0.9%)	9 (0.6%)	0.4
Hyperuricemia	1182	1659	59 (5.0%)	85 (5.1%)	0.9	0 (0%)	0 (0%)	
CREA increased	1484	2116	153 (10%)	179 (8.5%)	0.059	40 (2.7%)	40 (1.9%)	0.11
Hypoglycemia	645	879	88 (14%)	127 (14%)	0.7	0 (0%)	0 (0%)	
Hypercholesterolemia	198	351	13 (6.6%)	21 (6.0%)	0.8	0 (0%)	0 (0%)	
Hypertriglyceridemia	147	180	15 (10%)	37 (21%)	0.011	2 (1.4%)	0 (0%)	0.2

^a)^ Number of people who completed the follow‐up of data collection for this indicator;

^b)^ Severity grades were defined according to the National Cancer Institute Common Terminology Criteria for Adverse Events (CTCAE), version 5.0.

## 3 Discussion

Due to changes in epidemic prevention policies, large‐scale SARS‐CoV‐2 infections have occurred intermittently in China since December 2022. Among the patients admitted to the hospital, elderly patients account for a large proportion. We conducted the large‐scale, multicenter, real‐world study to evaluate the efficacy and safety of Azvudine in elderly patients with COVID‐19. We observed that compared with controls, patients receiving Azvudine can significantly reduce the risk of all‐cause death, especially for patients concomitant antibiotics. However, we did not observe a significant reduction in the risk of composite disease progression in Azvudine group compared with control group. Three sensitivity analyzes showed the same results, further confirming the reliability of our results.

The elderly are a special group of COVID‐19 patients, and the proportion of severe illness and death caused by SARS‐CoV‐2 infection is the highest among all groups of all ages. There may be three main reasons for this: (1) the elderly have low immunity and are susceptible to many diseases;^[^ ^8^ ^]^ (2) after being infected with viruses, the incidence of autoimmunity increases and inflammatory reactions are more likely to occur;^[^ ^9^ ^]^ (3) elderly patients are often accompanied by multiple diseases and repeated use of drugs. Infection with viruses can make the symptoms of the original disease more severe.^[^ ^10^ ^]^ Older adults need to be more careful in selecting antiviral medications due to limitations in complications and drug interactions.

During the first wave of large‐scale Omicron infections in China in December 2022, Azvudine, Molnupiravir, and Nirmatrelvir/ritonavir were the priority antiviral drugs recommended by “COVID‐19 diagnosis and treatment plan (trial version 9 or version 10)”, glucocorticoid and Tocilizumab were the recommended immunotherapy drugs for severe and critical severe patients. At present, there is still controversy about whether Paxlovid can treat elderly patients with COVID‐19. Carlos K H Wong et al. found that early oral administration of Paxlovid in Hong Kong elderly patients can reduce the risk of death and hospitalization.^[^ ^11^ ^]^ One study included veterans over 65 years old with mild to moderate COVID‐19. The results showed that Paxlovid intervention was associated with lower 30‐day hospitalization or mortality risks compared with no Paxlovid.^[^ ^12^ ^]^ However, Shuxia Wang et al. observed that Paxlovid was not associated with significant clinical benefit in elderly severe patients with COVID‐19, including composite outcomes and all‐cause death.^[^ ^13^ ^]^ On top of that, Paxlovid is expensive and in short supply, making it inaccessible to patients in developing countries. Moreover, ritonavir, as one of the components of Paxlovid, is a CYP3A inhibitor, which can impair the detoxification and metabolism of many drugs and cause serious adverse events.^[^ ^14^ ^]^ Molnupiravir, a nucleoside analogue, approved for the treatment of mild ormoderate adult patients with high risk of progression to severe disease within 5 days of onset, could effectively reduce all‐cause death in both hospitalized or nonhospitalized COVID‐19 patients.^[^ ^15^ ^]^ Additionally, the similar protect effect was observed in elderly hospitalized patients infected with Omicron.^[^ ^15^, ^16^ ^]^ The results of WHO Rapid Evidence Appraisal for COVID‐19 Therapies Working Group showed that systemic corticosteroids reduced 28‐day all‐cause mortality in patients with severe COVID‐19 compared with routine care or placebo.^[^ ^17^ ^]^ As for Tocilizumab (a IL‐6 inhibitor), a meta‐analysis showed that tocilizumab group had a lower mortality compared with controls.^[^ ^18^ ^]^ Nevertheless, another meta‐analysis study found tocilizumab use may not be associated with a short‐term mortality benefit for hospitalized COVID‐19 patients, indicating more high‐quality evidence were needed.^[^ ^19^ ^]^ There is insufficient evidence for the effects of the two immunotherapy drugs in elderly.

Azvudine, as a dual‐target nucleoside drug, is the first anti‐SARS‐CoV‐2 oral small molecule drug independently developed in China. Oral administration of Azvudine can lead to the accumulation of the active form in the thymus of rats, inhibit SARS‐CoV‐2 replication, preserve thymic immune function, and rapidly cure COVID‐19.^[^ ^7b^ ^]^ Studies have shown that Azvudine can significantly shorten the nucleic acid negative time of COVID‐19 patients, reduce all‐cause mortality and composite disease progression.^[^ ^7^, ^20^ ^]^ On July 14, 2022, the National Medical Products Administration of China approved it for the treatment of COVID‐19, and one month later it was included in the medical reimbursement catalog by the National Medical Insurance Administration of China.^[^ ^21^ ^]^ Nevertheless, there is also controversy over whether Azvudine can be used in the elder. Kaican Zong et al. found that Azvudine was effective in reducing all‐cause mortality in hospitalized COVID‐19 patients over 65 years of age by subgroup analysis.^[^ ^20a^ ^]^ Another single‐center study enrolled 128 Azvudine elderly recipients and 66 elderly controls, and found that compared with standard treatment, Azvudine did not reduce all‐cause mortality and composite outcomes in elderly severe patients.^[^ ^13^ ^]^ Zhou et al. revealed that compared with controls (n = 23), Azvudine (n = 14) were associated with a shorter time to nucleic acid negative conversion (NANC), length of hospital stay, and a smaller NANC rates.^[^ ^4b^ ^]^ Considering that these studies all have the shortcomings of small sample size, or single center, or selection bias and confounding bias, there is an urgent need to conduct multi‐center, large‐scale, real‐world studies to explore the effectiveness and safety of Azvudine in elderly COVID‐19 patients to assist clinical practice and decision making.

This study collected a total of 32 864 patients from 9 hospitals in Henan Province, which is the largest multicenter retrospective cohort study so far and could minimize the potential selection bias. After conducting a 1:1 PSM to control the confounding bias (such as vaccination doses, disease severity, BMI, gender, age, comorbidities and other potential factors affect the progress of COVID‐19), 4109 elderly COVID‐19 patients (aged over 60 years) who received Azvudine and 4109 elderly COVID‐19 patients who received standard treatment were included. The average age of the patients was 75.17 years old. COX regression results show that after adjusting for multiple baseline variables, elderly patients with Azvudine had a 33% lower risk of all‐cause death compared with controls (p < 0.001), which is consistent with the previous results.^[^ ^20a^ ^]^ Nonetheless, the results of COX regression analyzes did not observe that Azvudine can effectively reduce the composite disease progression in the elderly COVID‐19 population. To further enhance the reliability of the results, we conducted three sensitivity analyses by using different statistical analysis methods or populations, which also verified the above results. We speculate that because most of the elderly have underlying diseases, inflammation is easy to occur after infection, and the disease progresses rapidly, so it is necessary to take Azvudine as soon as possible. However, at that time, medical resources were so tight that they could not be admitted to hospital in time for treatment, and missed the best time for medication, which led to the inability of Azvudine to effectively control the progress of the disease.

Subgroup analysis showed that Azvudine could reduce all‐cause mortality without being affected by gender, severity, vaccination doses, concomitant systemic steroid, comorbidities. Nevertheless, different effects of Azvudine treatment for clinical outcomes were shown in people with or without antibiotics (p for interaction = 0.015). We found that Azvudine had a stronger protective effect on patients with antibiotics, significantly reducing the risk of all‐cause death by 43% and of composite disease progression by 21% versus standard therapy. We speculate that the possible reason is that the elderly have lower immunity and are more likely to have secondary or even triple bacterial infections. Therefore, Azvudine combined with antibiotics can play a better protective effect

There are few data on the safety of Azvudine in the treatment of COVID‐19, and previous results have mostly reported that Azvudine is associated with headache, dizziness, elevated transaminase, nausea and elevated D‐dimer levels.^[^ ^22^ ^]^ Although the Azvudine group had a higher incidence of graded ≥ 3 adverse events versus controls in this study, including lymphocyte count decreased (16% vs 12%, p < 0.001) and hypokalemia (10% vs 7.2%, p < 0.001), the safety of Azvudine in the treatment of elderly patients with COVID‐19 is acceptable. Nevertheless, this study failed to collect the symptom information of the patients, and could only show some of the possible adverse events. However, this research still provides a reference for the medication choice of elderly patients with COVID‐19.

The timing of antiviral drug administration is critical to clearing the SARS‐CoV‐2 and improving prognosis. Multiple studies and guidelines suggest that the applicable groups for Nirmatrelvir/ritonavir and Molnupiravir are adult patients with mild or moderate disease within 5 days of onset. For Azvudine, there is insufficient evidence to prove its optimal therapeutic window. A subgroup analysis of one study found that compared with the controls, oral Azvudine did not reduce the risk of all‐cause death and composite diseases in people with more than 5 days from onset to exposure (n = 214) (p > 0.05).^[^ ^7c^ ^]^ However, Zhou et al. found that compared with standard treatment, Azvudine also has potential therapeutic benefits for people with more than 5 days from onset to admission (n = 215).^[^ ^23^ ^]^ In addition, compared with Nirmatrelvir/ritonavir, Azvudine was found to significantly reduce composite disease progression in patients receiving the treatment beyond 5 days since onset,^[^ ^24^ ^]^ but this result was overturned in another study.^[^ ^25^ ^]^ Therefore, taken together, larger and more rigorous clinical trials still need to be conducted in the future to explore the optimal therapeutic window of Azvudine.

Due to the urgency of therapeutic regimen during an outbreak and the lengthy time of traditional drug development, drug repurposing—including screening approved drugs or clinical‐stage drug candidates originally developed for other human diseases—has been widely pursued to identify drugs that inhibit SARS‐CoV‐2 or mitigate the consequences of viral infection. Among them, reverse transcriptase (RT) inhibitors (RTI) are considered due to their core role in competitively inhibiting RNA‐dependent RNA polymerase (RDRP)‐mediated viral RNA synthesis, such as Azvudine or Tenofovir for human immunodeficiency virus, Sofosbuvir for hepatitis C virus, and Favipiravir for influenza virus.^[^ ^26^ ^]^ It has been proven that Azvudine, Molnupiravir, VV16, etc. have played an excellent role in fighting COVID‐19 as RTIs.^[^ ^27^ ^]^ Therefore, it is urgent to pay attention to research in this field. If a new RNA viral infection outbreak occurs again in the future, these inhibitors will be one of the main options for eliminating the infection burden.

## 4 Limitations

Our research also has some inevitable limitations. First, since this is a retrospective design trial, although we have controlled some potential confounding factors, we cannot completely avoid the selection bias that may exist in the retrospective study, nor can we avoid the possible impact of other confounding variables that have not been collected. Second, we only conducted a short‐term follow‐up of 31 days and did not evaluate the long‐term efficacy of Azvudine in the treatment of elderly COVID‐19. Third, BA.5.2 is the dominant strain in China during December 2022. Our study does not provide the data of Azvudine on other strain of SRAS‐CoV‐2. Whether Azvudine is effective against other strain still needs to be studied. Finally, the study included only hospitalized elderly COVID‐19 patients. As a result, the results of the study may not be extended to elderly outpatients. Although this study has some limitations, it still provides good evidence for accurately estimating the efficacy and safety of Azvudine in elderly patients with COVID‐19.

## 5 Conclusions 

Overall, this large‐scale, multicenter, retrospective study confirms the effectiveness and safety of Azvudine in reducing all‐cause death in COVID‐19 people aged over 60 years. Although there was no significant difference in the risk of composite disease progression, this study suggests that Azvudine can be used as an alternative antiviral therapy for elderly patients with COVID‐19, and can provide more choices and opportunities for clinical treatment decisions.

## 6 Experimental Section

### Study Design and Population

A muti‐center, retrospective cohort study of elderly patients were identified with COVID‐19 admitted to nine hospitals of Henan, China from December 5, 2022 and January 31, 2023, including the First Affiliated Hospital of Zhengzhou University, Henan Provincial Chest Hospital, Henan Infectious Disease Hospital, Luoyang Central Hospital, Nanyang Central Hospital, the Fifth People's Hospital of Anyang, Shangqiu Municipal Hospital, Guangshan County People's Hospital, Fengqiu County People's Hospital. Inclusion criteria were as follows: (1) patients were positive for SARS‐CoV‐2 infection through reverse transcription polymerase chain reaction; (2) aged ≥ 60 years; (3) obtained standard treatment or Azvudine plus standard treatment. Exclusion criteria were as follows: (1) patients aged <60 years; (2) pregnant women; (3) received other antiviral agents other than Azvudine.

Patient severity and treatment plan were performed according to COVID‐19 diagnosis and treatment plan (trial version 9 or version 10) published by the National Health Committee of China.^[^ ^5^ ^]^ Mild disease was defined as the main manifestation of upper respiratory tract infection. Moderate disease was defined as persistent high fever for >3 days or/and cough, shortness of breath, etc., but the respiratory rate (RR) was <30 times/min, the oxygen saturation at rest was > 93%, and characteristic symptoms of pneumonia caused by SARS‐CoV‐2 can be seen on imaging. Severe disease in this study was defined based on the criteria of severe and critical severe COVID‐19 in the guidelines, including respiratory rate ≥ 30 times/min, or resting oxygen saturation ≤ 93%, or PaO2/FiO2 ≤ 300 mmHg, or lung lesions progressing >50% at 24–48 hours, or the need for mechanical ventilation, or shock, or ICU monitoring.

All eligible patients were allocated to the control group (receiving standard treatment and without the use of any antiviral agents throughout the observation period), and Azvudine group (receiving standard treatment plus 5 mg Azvudine once daily for up to 14 days). All participants were observed from the date of confirmed SARS‐CoV‐2 infection until the date of outcome events or 31 days.

### Ethic

This study follows the Declaration of Helsinki and STROBE guidelines, and has been approved by the Institutional Review Board and Ethics Committee of the First Affiliated Hospital of Zhengzhou University (Approval No. 2023‐KY‐0865‐001). The study was registered with ClinicalTrials.gov (NCT06349655). Since all patients were anonymous, individual informed consent was not required.

### Data Collection and Outcomes

Demographic characteristics, dates of hospital admissions, admission to ICU, registered death, diagnoses, prescription and drug dispensing records, procedures, imaging data, and laboratory tests were collected from the electronic medical records of patients from nine hospitals in Henan Province.

The baseline variables at admission of patients included age, sex, BMI, severity, vaccination doses, concomitant antibiotics or systemic steroid (defined as “No” and “Yes” based on whether antibiotics or systemic steroid has been used within one day of admission), time from diagnosis to treatment exposure (defined as “>5 days” or “0–5 days”), clinical laboratory indicators (neutrophil, lymphocyte, glucose, high‐density lipoprotein, low‐density lipoprotein, alanine aminotransferase, aspartate aminotransferase, creatinine, glomerular filtration rate, C–reactive protein, procalcitonin, prothrombin time, activated partial thromboplastin time, cholesterol, triglyceride, alkaline phosphatase, gamma‐glutamyl transpeptidase, albumin, and total bilirubin), and comorbidity (diabetes, hypertension, liver diseases, cardio‐cerebral diseases, kidney diseases, primary malignant tumor, chronic respiratory diseases, and autoimmune diseases).

The primary outcome was all‐cause death. The secondary outcomes was composite disease progression, defined as progression to severe disease in mild or moderate patients, and the occurrence of death. The safety outcome was overall adverse events (AEs), and AEs with grade ≥ 3. AEs was defined according to the Common Terminology Criteria for Adverse Events, Version 5.0.^[^ ^28^ ^]^ The starting point for safety data collection was after drug use and the end point was the fifth half‐life after the last dose. When the grade of the same adverse event changes during the observation period, the most serious grade was recorded.

### Statistical Analysis

The logistic regression model combined with greedy match at a ratio of 1:1 was used to reduce the impact of confounding factors, including all baseline variables. After PSM, the balance of baseline variables were calculated between control and Azvudine groups using the p value, with p > 0.05 and SMD < 10% meaning the best balance between two groups.

The cumulative event curves were generated by the Kaplan‐Meier method and the survival difference between groups was assessed by the log‐rank test. The HRs with 95% CI were calculated by cox proportional hazards regression models, with adjusting for all baseline covariates. The proportional hazards (PH) assumption was assessed using Schoenfeld residuals, and a non‐proportional hazards Cox regression model based on time dependent covariate was constructed if the PH assumption does not hold.

To understand in which specific patients Azvudine would be more effective, subgroup analyzes stratified by gender, age, severity, concomitant therapy, time from diagnosis was conducted to treatment exposure, diabetes, hypertension, liver diseases, cardio‐cerebral diseases, kidney diseases and primary malignant tumor. Three sensitive analysis was conducted to test the robustness of the findings. First, the average was used to fill in missing data. Second, a probit regression model was used to perform a 1:1 greedy match. Third, because it takes a certain amount of time for the drug to work after taking it, patients were excluded who were discharged or died on the same day after taking the drug.

The 2‐sided p value < 0.05 was defined as statistical significance. Continuous variables were expressed as mean (standard deviation) if normally distributed or as median (interquartile range) if non‐normally distributed. Categorical variables were expressed as frequency (percentage). To compare the difference between groups, independent t test was used if continuous variables was normally distributed or Mann–Whitney U test if non‐normally distributed, and used the Chi‐square test for categorical variables. Multiple imputations were used to impute the missing values. All data in this study were analyzed by R version 4.0.3.

## Conflict of Interest

The authors declare no conflict of interest.

## Author Contributions

R.S., H.W., J.S., M.Y., S.Z., and X.H. contributed equally to this work. H.L. and Z.R. conceived and designed the study. R.S., Q.K., X.H., S.Z., Z.S., H.L., G.L., Y. Y., G.L., D.Z., N.H., and S.L. managed the patients. J.S., B.Y., M.Y., H.W., S.Z., Z.S., L.L., Y.Z., and M.C. acquired the data. R.S., M.Y., J.S., and H.W. analyzed the data. R.S., H.W., S.Z., X.H., and Z.S. wrote the manuscript. All authors reviewed and approved the manuscript.

## Supporting information



Supporting Information

## Data Availability

The data that support the findings of this study are available from the corresponding author upon reasonable request.
