# Supplementary material for: Effectiveness and Safety of Oral Azvudine for Elderly Hospitalized Patients With COVID‐19: A Multicenter, Retrospective, Real‐World Study
Source: Adv Sci (Weinh). 2025 Feb 11;12(13):2404450. doi: 10.1002/advs.202404450 (PMC11967827; doi:10.1002/advs.202404450)
Supplement: Supplementary file 1 — Supporting Information [file ADVS-12-2404450-s001.docx]

**Supplementary Material**

**Effectiveness and safety of oral Azvudine for elderly hospitalized patients with COVID-19: A multicenter, retrospective, real‐world study**

**Authors**

Ranran Sun ^1†^，Haiyu Wang ^1†^, Junyi Sun ^1†^, Mengzhao Yang ^1†^, Shixi Zhang ^2†^, Xinjun Hu ^3†^, Bo Yu ^4^, Zhan Song ^5^, Na Han ^1^, Hong Luo ^6^, Ming Cheng ^7^, Guangming Li ^8^, Guotao Li ^9^, Yiqiang Yuan ^10^, Lili Liang ^10^, Yanyang Zhang ^11^, Donghua Zhang ^12^, Silin Li ^13^, Quancheng Kan ^14^, Hongxia Liang ^1^*, Zhigang Ren ^1^*

**Supplementary Figures**


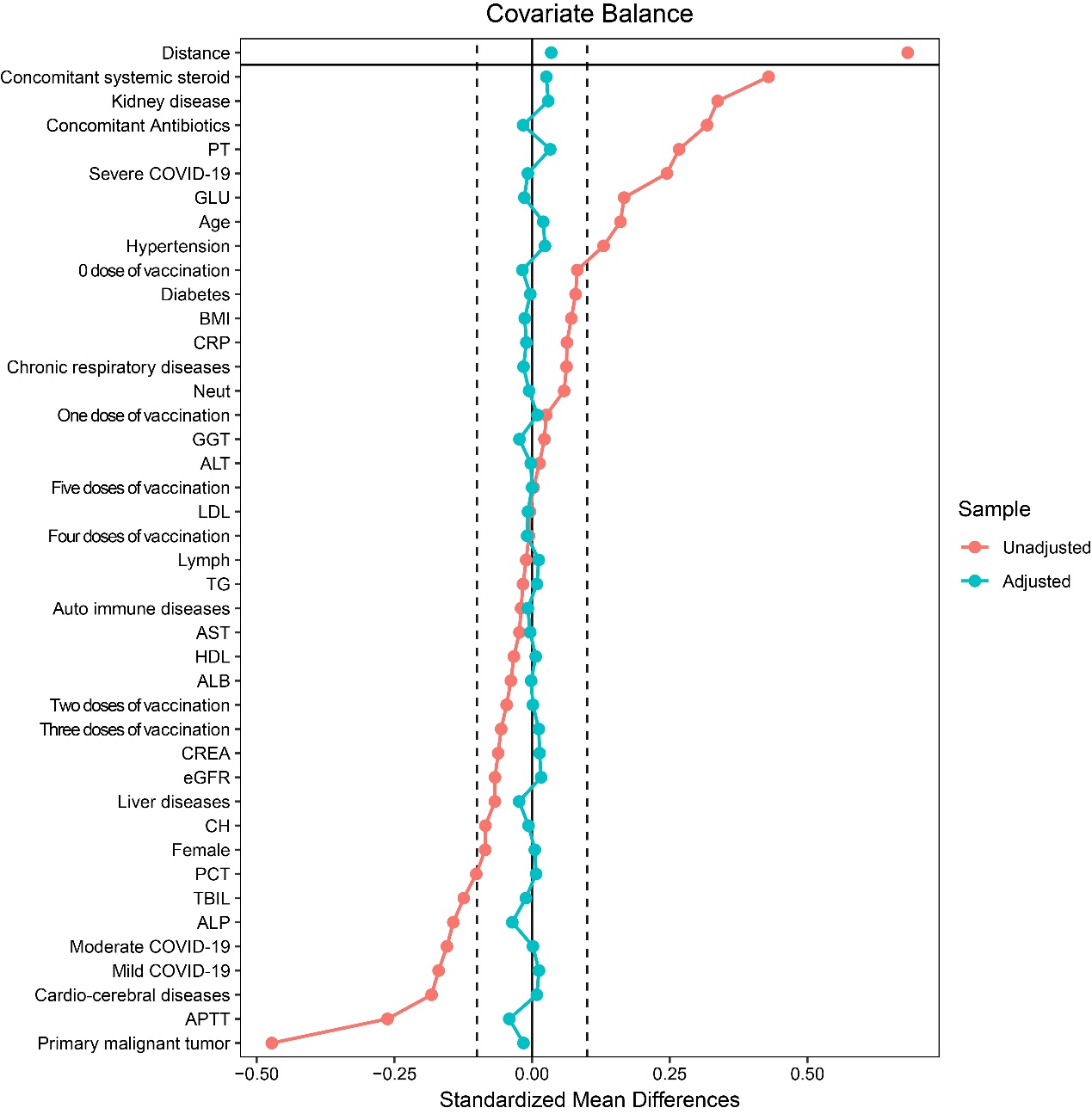


**Figure S1. Baseline characteristics before and after propensity score matching.**


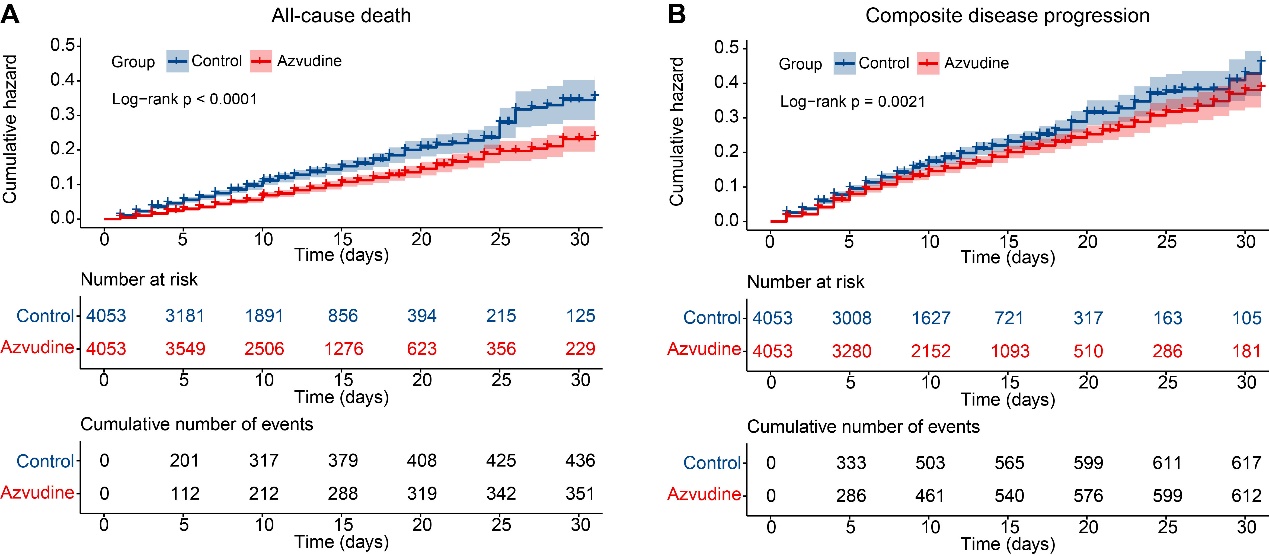


**Figure S2.** **Kaplan–Meier curves of patients receiving standard treatment versus Azvudine treatment in the sensitivity analysis where the missing data was filled up with the mean value.** Cumulative hazard of all‐cause death (A) and composite disease progression (B).


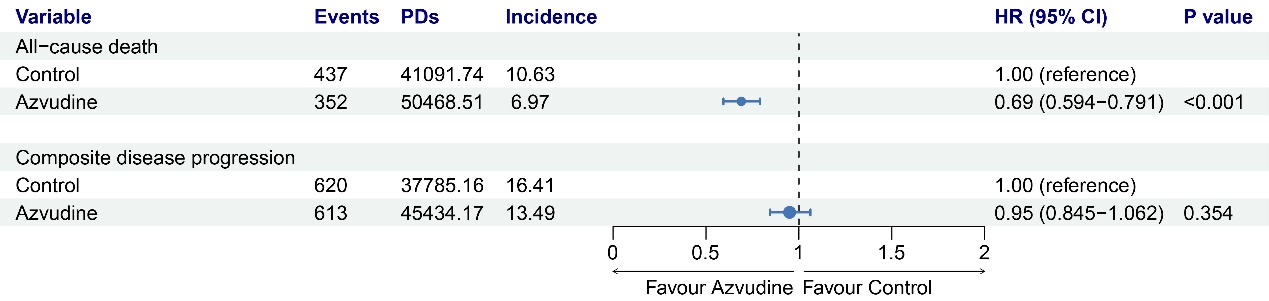


**Figure S3.** **Multivariate Cox proportional hazards regression analysis of all-cause death and composite disease progression in patients receiving standard treatment and Azvudine treatment in the sensitivity analysis where the missing data was filled up with the mean value.** Adjusted for all baseline covariates in Table S1. HR: Hazard Ratio; 95% CI: 95% confidence interval. PDs: Person-days. Incidence: events/per 1000 PDs.


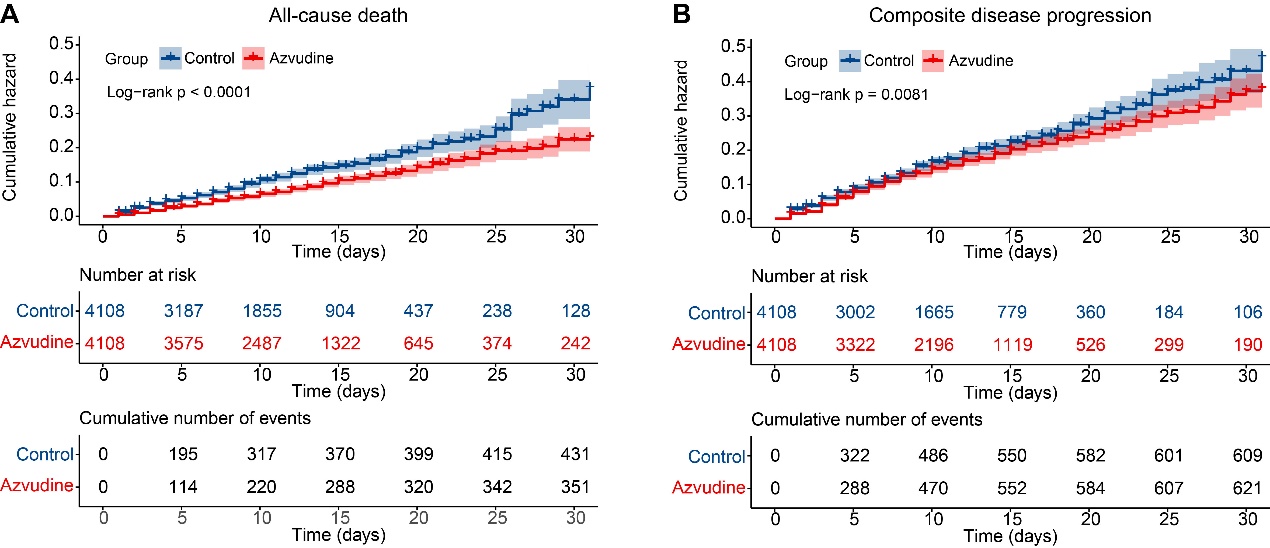


**Figure S4.** **Kaplan–Meier curves of patients receiving standard treatment versus Azvudine treatment in the sensitivity analysis where propensity score matching was performed using probit method.** Cumulative hazard of all‐cause death (A) and composite disease progression (B).


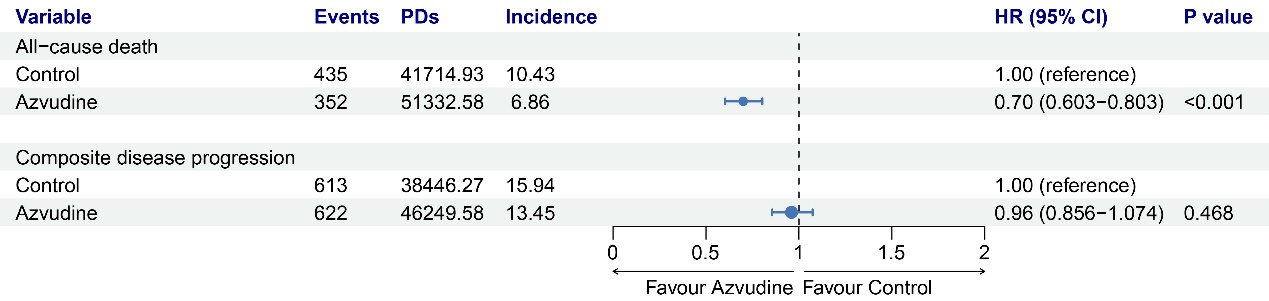


**Figure S5.** **Multivariate Cox proportional hazards regression analysis of all-cause death and composite disease progression in patients receiving standard treatment and Azvudine treatment in the sensitivity analysis where propensity score matching was performed using probit method.** Adjusted for all baseline covariates in Table S2. HR: Hazard Ratio; 95% CI: 95% confidence interval. PDs: Person-days. Incidence: events/per 1000 PDs.


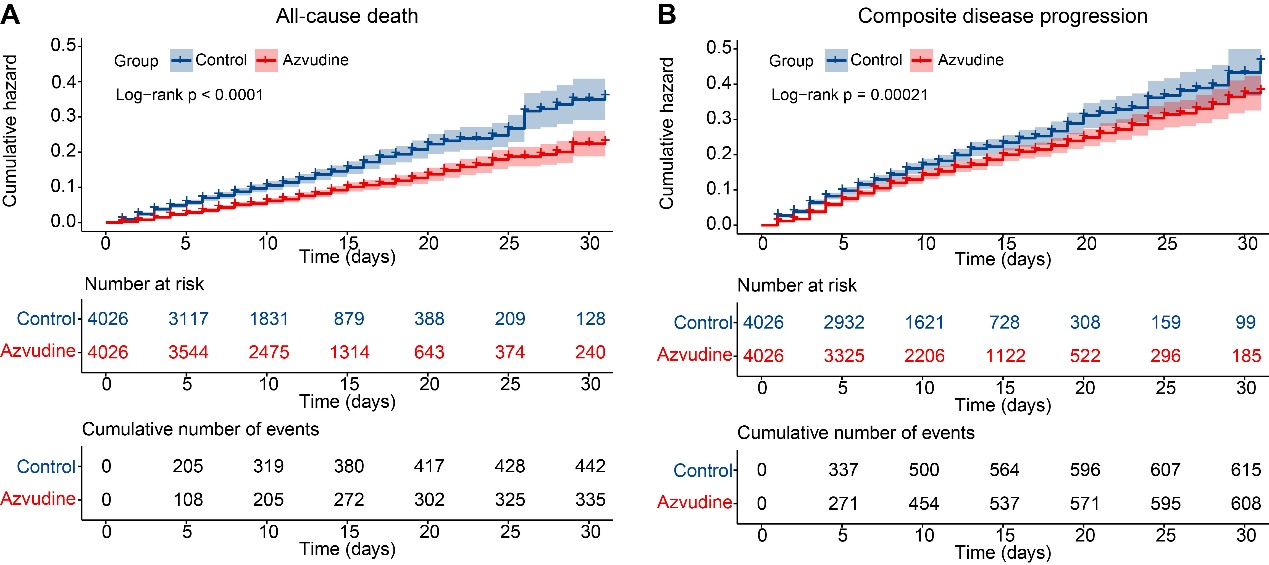


**Figure S6.** **Kaplan–Meier curves of patients receiving standard treatment versus Azvudine treatment in the sensitivity analysis where patients who discharged within one day after receiving antiviral treatment were excluded.** Cumulative hazard of all‐cause death (A) and composite disease progression (B).


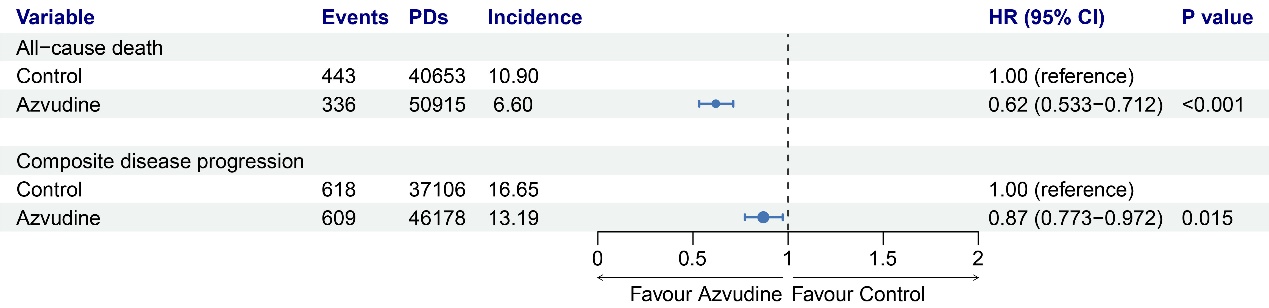


**Figure S7.** **Multivariate Cox proportional hazards regression analysis of all-cause death and composite disease progression in patients receiving standard treatment and Azvudine treatment in the sensitivity analysis where patients who discharged within one day after receiving antiviral treatment were excluded.** Adjusted for all baseline covariates in Table S3. HR: Hazard Ratio; 95% CI: 95% confidence interval. PDs: Person-days. Incidence: events/per 1000 PDs.

**Supplemental Tables**

**Table S1. Baseline characteristics of elderly patients with COVID-19 before and after propensity score matching in the sensitivity analysis where the missing data was filled up with the mean value**

| **Characteristics** | **Before matching** | | |  | **After 1:1 matching** | | |
| --- | --- | --- | --- | --- | --- | --- | --- |
|  | **Control**  **(n=15518)** | **Azvudine (n=4245)** | **P value** |  | **Control (n=4053)** | **Azvudine (n=4053)** | **P value** |
| **Age, mean (SD), year** | 73.93 (8.66) | 75.28 (8.55) | **<0.001** |  | 75.42 (8.84) | 75.28 (8.55) | **0.468** |
| **Gender, n (%)** |  |  | **<0.001** |  |  |  | **1.0** |
| **Male** | 8903 (57.4) | 2613 (61.6) |  |  | 2478 (61.1) | 2477 (61.1) |  |
| **Female** | 6615 (42.6) | 1632 (38.4) |  |  | 1575 (38.9) | 1576 (38.9) |  |
| **BMI, mean (SD), kg/m^2^** | 23.38 (2.34) | 23.58 (2.00) | **<0.001** |  | 23.62 (2.36) | 23.58 (2.02) | **0.496** |
| **Severity at admission, n (%)** |  |  | **<0.001** |  |  |  | **0.128** |
| **Mild** | 1318 (8.5) | 206 (4.9) |  |  | 224 (5.5) | 204 (5.0) |  |
| **Moderate** | 11722 (75.5) | 2901 (68.3) |  |  | 2718 (67.1) | 2802 (69.1) |  |
| **Severe^a^** | 2478 (16.0) | 1138 (26.8) |  |  | 1111 (27.4) | 1047 (25.8) |  |
| **Vaccination doses, n (%)** |  |  | **<0.001** |  |  |  | **0.845** |
| **0 dose** | 4077 (26.3) | 1276 (30.1) |  |  | 1227 (30.3) | 1213 (29.9) |  |
| **1 dose** | 957 (6.2) | 292 (6.9) |  |  | 271 (6.7) | 280 (6.9) |  |
| **2 doses** | 2237 (14.4) | 547 (12.9) |  |  | 505 (12.5) | 528 (13.0) |  |
| **3 doses** | 8050 (51.9) | 2079 (49.0) |  |  | 1996 (49.2) | 1982 (48.9) |  |
| **4 doses** | 194 (1.3) | 50 (1.2) |  |  | 54 (1.3) | 49 (1.2) |  |
| **5 doses** | 3 (0.0) | 1 (0.0) |  |  | 0 (0.0) | 1 (0.0) |  |
| **Concomitant systemic steroid, n (%)** |  |  | **<0.001** |  |  |  | **0.291** |
| **No** | 11820 (76.2) | 2326 (54.8) |  |  | 2364 (58.3) | 2316 (57.1) |  |
| **Yes** | 3698 (23.8) | 1919 (45.2) |  |  | 1689 (41.7) | 1737 (42.9) |  |
| **Concomitant antibiotics, n (%)** |  |  | **<0.001** |  |  |  | **0.607** |
| **No** | 9200 (59.3) | 1848 (43.5) |  |  | 1794 (44.3) | 1818 (44.9) |  |
| **Yes** | 6318 (40.7) | 2397 (56.5) |  |  | 2259 (55.7) | 2235 (55.1) |  |
| **Comorbidities, n (%)** |  |  |  |  |  |  |  |
| **Diabetes** | 3663 (23.6) | 1151 (27.1) | **<0.001** |  | 1134 (28.0) | 1107 (27.3) | **0.518** |
| **Hypertension** | 6360 (41.0) | 2015 (47.5) | **<0.001** |  | 1902 (46.9) | 1905 (47.0) | **0.964** |
| **Liver diseases** | 1600 (10.3) | 358 (8.4) | **<0.001** |  | 369 (9.1) | 357 (8.8) | **0.669** |
| **Cardio-cerebral diseases** | 6937 (44.7) | 1526 (35.9) | **<0.001** |  | 1540 (38.0) | 1524 (37.6) | **0.731** |
| **Kidney diseases** | 1953 (12.6) | 1174 (27.7) | **<0.001** |  | 983 (24.3) | 1007 (24.8) | **0.553** |
| **Primary malignant tumor** | 3415 (22.0) | 369 (8.7) | **<0.001** |  | 343 (8.5) | 369 (9.1) | **0.327** |
| **Chronic respiratory diseases** | 2876 (18.5) | 895 (21.1) | **<0.001** |  | 844 (20.8) | 852 (21.0) | **0.848** |
| **Autoimmune diseases** | 440 (2.8) | 107 (2.5) | **0.291** |  | 108 (2.7) | 100 (2.5) | **0.623** |
| **Laboratory parameters, mean (SD)** |  |  |  |  |  |  |  |
| **Neutrophil, ×10^9^/L** | 5.66 (4.54) | 5.92 (3.87) | **0.001** |  | 5.93 (3.85) | 5.90 (3.85) | **0.698** |
| **Lymphocyte, ×10^9^/L** | 1.49 (13.28) | 1.36 (16.56) | **0.588** |  | 1.37 (11.44) | 1.38 (16.95) | **0.977** |
| **Glucose, mmol/L** | 7.31 (3.32) | 8.03 (3.67) | **<0.001** |  | 7.97 (4.11) | 7.94 (3.56) | **0.761** |
| **High-density lipoprotein, mmol/L** | 1.24 (1.94) | 1.22 (1.78) | **0.533** |  | 1.26 (2.22) | 1.23 (1.82) | **0.518** |
| **Low-density lipoprotein, mmol/L** | 2.44 (1.98) | 2.38 (1.82) | **0.072** |  | 2.41 (2.24) | 2.39 (1.85) | **0.648** |
| **Alanine aminotransferase, IU/L** | 33.69 (80.98) | 34.89 (65.15) | **0.373** |  | 35.76 (107.15) | 34.59 (63.93) | **0.55** |
| **Aspartate aminotransferase, IU/L** | 41.85 (107.93) | 40.40 (70.05) | **0.408** |  | 40.51 (88.79) | 40.09 (70.82) | **0.815** |
| **Creatine, μmol/L** | 100.18 (188.60) | 92.74 (112.99) | **0.014** |  | 94.97 (101.24) | 93.10 (114.12) | **0.435** |
| **Glomerular filtration rate, ml/min** | 105.92 (81.16) | 100.39 (14.84) | **<0.001** |  | 100.08 (40.77) | 100.21 (15.12) | **0.845** |
| **C-reactive protein, mg/L** | 49.62 (42.67) | 53.92 (50.23) | **<0.001** |  | 52.72 (47.80) | 53.40 (49.94) | **0.526** |
| **Procalcitonin, ng/ml** | 1.88 (7.42) | 1.26 (4.90) | **<0.001** |  | 1.38 (4.80) | 1.26 (4.98) | **0.278** |
| **Prothrombin time, s** | 14.67 (6.09) | 17.58 (9.64) | **<0.001** |  | 16.53 (8.13) | 16.89 (9.06) | **0.061** |
| **Activated partial thromboplastin time, s** | 29.36 (8.86) | 26.11 (9.93) | **<0.001** |  | 27.05 (8.02) | 26.68 (9.74) | **0.061** |
| **Cholesterol, mmol/L** | 4.34 (3.44) | 4.14 (1.82) | **<0.001** |  | 4.18 (2.35) | 4.15 (1.85) | **0.554** |
| **Triglyceride, mmol/L** | 1.75 (3.53) | 1.60 (1.88) | **0.007** |  | 1.67 (2.43) | 1.61 (1.88) | **0.233** |
| **Alkaline phosphatase, IU/L** | 91.08 (66.60) | 82.06 (48.65) | **<0.001** |  | 82.80 (38.56) | 82.53 (48.77) | **0.778** |
| **Gamma-glutamyl transpeptidase, IU/L** | 52.58 (79.53) | 54.11 (67.52) | **0.252** |  | 53.30 (79.42) | 53.59 (63.51) | **0.857** |
| **Albumin, g/L** | 38.37 (18.13) | 37.05 (31.57) | **<0.001** |  | 37.44 (28.61) | 37.31 (32.27) | **0.845** |
| **Total bilirubin, μmol/L** | 14.35 (24.36) | 12.56 (10.71) | **<0.001** |  | 12.63 (9.66) | 12.53 (10.81) | **0.679** |

^a^: Patients with severe disease in this study are defined as patients with severe and critical disease in the guidelines.

**Table S2. Baseline characteristics of elderly patients with COVID-19 before and after propensity score matching using probit method**

| **Characteristics** | **Before matching** | | |  | **After 1:1 matching** | | |
| --- | --- | --- | --- | --- | --- | --- | --- |
|  | **Control**  **(n=15518)** | **Azvudine (n=4245)** | **P value** |  | **Control (n=4108)** | **Azvudine (n=4108)** | **P value** |
| **Age, mean (SD), year** | 73.92 (8.72) | 75.29 (8.55) | **<0.001** |  | 75.32 (8.84) | 75.26 (8.55) | **0.751** |
| **Gender, n (%)** |  |  | **<0.001** |  |  |  | **0.667** |
| **Male** | 8913 (57.4) | 2614 (61.6) |  |  | 2529 (61.6) | 2509 (61.1) |  |
| **Female** | 6605 (42.6) | 1631 (38.4) |  |  | 1579 (38.4) | 1599 (38.9) |  |
| **BMI, mean (SD), kg/m^2^** | 23.37 (3.83) | 23.64 (3.84) | **<0.001** |  | 23.58 (3.85) | 23.62 (3.81) | **0.664** |
| **Severity at admission, n (%)** |  |  | **<0.001** |  |  |  | **0.847** |
| **Mild** | 1319 (8.5) | 206 (4.9) |  |  | 200 (4.9) | 202 (4.9) |  |
| **Moderate** | 11721 (75.5) | 2901 (68.3) |  |  | 2818 (68.6) | 2839 (69.1) |  |
| **Severe^a^** | 2478 (16.0) | 1138 (26.8) |  |  | 1090 (26.5) | 1067 (26.0) |  |
| **Vaccination doses, n (%)** |  |  | **0.664** |  |  |  | **0.876** |
| **0 dose** | 4102 (26.4) | 1282 (30.2) |  |  | 1250 (30.4) | 1231 (30.0) |  |
| **1 dose** | 953 (6.1) | 288 (6.8) |  |  | 275 (6.7) | 278 (6.8) |  |
| **2 doses** | 2221 (14.3) | 542 (12.8) |  |  | 539 (13.1) | 530 (12.9) |  |
| **3 doses** | 8043 (51.8) | 2081 (49.0) |  |  | 1988 (48.4) | 2018 (49.1) |  |
| **4 doses** | 196 (1.3) | 51 (1.2) |  |  | 56 (1.4) | 50 (1.2) |  |
| **5 doses** | 3 (0.0) | 1 (0.0) |  |  | 0 (0.0) | 1 (0.0) |  |
| **Concomitant systemic steroid, n (%)** |  |  | **<0.001** |  |  |  | **0.327** |
| **No** | 11820 (76.2) | 2326 (54.8) |  |  | 2366 (57.6) | 2321 (56.5) |  |
| **Yes** | 3698 (23.8) | 1919 (45.2) |  |  | 1742 (42.4) | 1787 (43.5) |  |
| **Concomitant antibiotics, n (%)** |  |  | **<0.001** |  |  |  | **0.912** |
| **No** | 9200 (59.3) | 1848 (43.5) |  |  | 1818 (44.3) | 1824 (44.4) |  |
| **Yes** | 6318 (40.7) | 2397 (56.5) |  |  | 2290 (55.7) | 2284 (55.6) |  |
| **Comorbidities, n (%)** |  |  |  |  |  |  |  |
| **Diabetes** | 3663 (23.6) | 1151 (27.1) | **<0.001** |  | 1124 (27.4) | 1121 (27.3) | **0.961** |
| **Hypertension** | 6360 (41.0) | 2015 (47.5) | **<0.001** |  | 1936 (47.1) | 1923 (46.8) | **0.791** |
| **Liver diseases** | 1600 (10.3) | 358 (8.4) | **<0.001** |  | 391 (9.5) | 357 (8.7) | **0.206** |
| **Cardio-cerebral diseases** | 6937 (44.7) | 1526 (35.9) | **<0.001** |  | 1595 (38.8) | 1521 (37.0) | **0.097** |
| **Kidney diseases** | 1953 (12.6) | 1174 (27.7) | **<0.001** |  | 1043 (25.4) | 1050 (25.6) | **0.879** |
| **Primary malignant tumor** | 3415 (22.0) | 369 (8.7) | **<0.001** |  | 371 (9.0) | 369 (9.0) | **0.969** |
| **Chronic respiratory diseases** | 2876 (18.5) | 895 (21.1) | **<0.001** |  | 878 (21.4) | 852 (20.7) | **0.499** |
| **Autoimmune diseases** | 440 (2.8) | 107 (2.5) | **0.291** |  | 102 (2.5) | 101 (2.5) | **1** |
| **Laboratory parameters, mean (SD)** |  |  |  |  |  |  |  |
| **Neutrophil, ×10^9^/L** | 5.67 (5.16) | 5.91 (4.05) | **0.006** |  | 5.89 (4.10) | 5.91 (4.06) | **0.751** |
| **Lymphocyte, ×10^9^/L** | 1.52 (15.47) | 1.34 (16.57) | **0.512** |  | 1.38 (14.79) | 1.35 (16.84) | **0.937** |
| **Glucose, mmol/L** | 7.32 (3.89) | 8.01 (4.13) | **<0.001** |  | 8.01 (4.55) | 7.96 (4.11) | **0.637** |
| **High-density lipoprotein, mmol/L** | 1.25 (2.68) | 1.18 (2.08) | **0.122** |  | 1.21 (2.32) | 1.18 (2.11) | **0.662** |
| **Low-density lipoprotein, mmol/L** | 2.43 (2.50) | 2.42 (2.75) | **0.81** |  | 2.40 (2.32) | 2.41 (2.72) | **0.864** |
| **Alanine aminotransferase, IU/L** | 33.55 (85.23) | 34.44 (66.23) | **0.532** |  | 34.45 (82.41) | 34.04 (65.84) | **0.802** |
| **Aspartate aminotransferase, IU/L** | 42.37 (122.06) | 40.37 (87.11) | **0.317** |  | 40.21 (81.56) | 40.08 (88.16) | **0.946** |
| **Creatine, μmol/L** | 99.72 (194.99) | 92.40 (118.86) | **0.02** |  | 94.40 (123.72) | 92.90 (120.63) | **0.58** |
| **Glomerular filtration rate, ml/min** | 105.54 (144.45) | 97.34 (121.73) | **0.001** |  | 95.81 (117.45) | 97.70 (123.02) | **0.476** |
| **C-reactive protein, mg/L** | 49.49 (62.00) | 53.56 (64.38) | **<0.001** |  | 51.57 (63.36) | 53.33 (64.51) | **0.212** |
| **Procalcitonin, ng/ml** | 1.90 (10.48) | 1.20 (6.93) | **<0.001** |  | 1.24 (6.90) | 1.21 (6.99) | **0.853** |
| **Prothrombin time, s** | 14.67 (8.14) | 17.58 (10.90) | **<0.001** |  | 16.98 (10.67) | 17.08 (10.34) | **0.667** |
| **Activated partial thromboplastin time, s** | 29.31 (10.98) | 26.21 (11.79) | **<0.001** |  | 27.11 (11.45) | 26.61 (11.74) | **0.051** |
| **Cholesterol, mmol/L** | 4.37 (4.48) | 4.11 (3.08) | **<0.001** |  | 4.11 (2.30) | 4.12 (3.13) | **0.84** |
| **Triglyceride, mmol/L** | 1.72 (4.58) | 1.63 (5.12) | **0.302** |  | 1.61 (3.78) | 1.64 (5.17) | **0.741** |
| **Alkaline phosphatase, IU/L** | 91.10 (72.94) | 82.53 (59.88) | **<0.001** |  | 83.82 (53.64) | 82.60 (60.20) | **0.331** |
| **Gamma-glutamyl transpeptidase, IU/L** | 52.27 (91.45) | 54.19 (85.68) | **0.22** |  | 52.51 (86.32) | 53.39 (82.40) | **0.636** |
| **Albumin, g/L** | 38.36 (20.68) | 37.13 (31.94) | **0.003** |  | 37.53 (21.01) | 37.30 (32.42) | **0.711** |
| **Total bilirubin, μmol/L** | 14.42 (26.12) | 12.64 (14.33) | **<0.001** |  | 12.73 (12.04) | 12.60 (14.47) | **0.658** |

^a^: Patients with severe disease in this study are defined as patients with severe and critical disease in the guidelines.

**Table S3. Baseline characteristics before and after propensity score matching, in the sensitivity analysis where elderly COVID-19 patients who discharged within one day after receiving azvudine treatment were excluded**

| **Characteristics** | **Before matching** | | |  | **After 1:1 matching** | | |
| --- | --- | --- | --- | --- | --- | --- | --- |
|  | **Control**  **(n=15305)** | **Azvudine (n=4147)** | **P value** |  | **Control (n=4026)** | **Azvudine (n=4026)** | **P value** |
| **Age, mean (SD), year** | 73.92 (8.72) | 75.33 (8.55) | <0.001 |  | 75.39 (8.87) | 75.31 (8.54) | 0.676 |
| **Gender, n (%)** |  |  | **<0.001** |  |  |  | **0.801** |
| **Male** | 8766 (57.3) | 2555 (61.6) |  |  | 2476 (61.5) | 2464 (61.2) |  |
| **Female** | 6539 (42.7) | 1592 (38.4) |  |  | 1550 (38.5) | 1562 (38.8) |  |
| **BMI, mean (SD), kg/m^2^** | 23.40 (3.82) | 23.54 (3.90) | 0.036 |  | 23.54 (3.90) | 23.55 (3.91) | 0.918 |
| **Severity at admission, n (%)** |  |  | **<0.001** |  |  |  | **0.556** |
| **Mild** | 1318 (8.6) | 202 (4.9) |  |  | 183 (4.5) | 201 (5.0) |  |
| **Moderate** | 11534 (75.4) | 2832 (68.3) |  |  | 2764 (68.7) | 2772 (68.9) |  |
| **Severe^a^** | 2453 (16.0) | 1113 (26.8) |  |  | 1079 (26.8) | 1053 (26.2) |  |
| **Vaccination doses, n (%)** |  |  | **<0.001** |  |  |  | **NA** |
| **0 dose** | 4009 (26.2) | 1245 (30.0) |  |  | 1205 (29.9) | 1208 (30.0) |  |
| **1 dose** | 942 (6.2) | 282 (6.8) |  |  | 249 (6.2) | 271 (6.7) |  |
| **2 doses** | 2192 (14.3) | 526 (12.7) |  |  | 546 (13.6) | 513 (12.7) |  |
| **3 doses** | 7963 (52.0) | 2044 (49.3) |  |  | 1973 (49.0) | 1985 (49.3) |  |
| **4 doses** | 196 (1.3) | 50 (1.2) |  |  | 53 (1.3) | 49 (1.2) |  |
| **5 doses** | 3 (0.0) | 0 (0.0) |  |  | 0 (0.0) | 0 (0.0) |  |
| **Concomitant systemic steroid, n (%)** |  |  | **<0.001** |  |  |  | **0.431** |
| **No** | 11613 (75.9) | 2276 (54.9) |  |  | 2312 (57.4) | 2276 (56.5) |  |
| **Yes** | 3692 (24.1) | 1871 (45.1) |  |  | 1714 (42.6) | 1750 (43.5) |  |
| **Concomitant antibiotics, n (%)** |  |  | **<0.001** |  |  |  | **0.334** |
| **No** | 9111 (59.5) | 1800 (43.4) |  |  | 1741 (43.2) | 1785 (44.3) |  |
| **Yes** | 6194 (40.5) | 2347 (56.6) |  |  | 2285 (56.8) | 2241 (55.7) |  |
| **Comorbidities, n (%)** |  |  |  |  |  |  |  |
| **Diabetes** | 3663 (23.9) | 1124 (27.1) | <0.001 |  | 1124 (27.9) | 1101 (27.3) | 0.584 |
| **Hypertension** | 6360 (41.6) | 1974 (47.6) | <0.001 |  | 1897 (47.1) | 1896 (47.1) | 1 |
| **Liver diseases** | 1597 (10.4) | 355 (8.6) | <0.001 |  | 378 (9.4) | 355 (8.8) | 0.394 |
| **Cardio-cerebral diseases** | 6910 (45.1) | 1504 (36.3) | <0.001 |  | 1521 (37.8) | 1504 (37.4) | 0.713 |
| **Kidney diseases** | 1953 (12.8) | 1149 (27.7) | <0.001 |  | 991 (24.6) | 1034 (25.7) | 0.281 |
| **Primary malignant tumor** | 3415 (22.3) | 359 (8.7) | <0.001 |  | 365 (9.1) | 359 (8.9) | 0.846 |
| **Chronic respiratory diseases** | 2875 (18.8) | 880 (21.2) | <0.001 |  | 863 (21.4) | 854 (21.2) | 0.828 |
| **Autoimmune diseases** | 440 (2.9) | 102 (2.5) | 0.165 |  | 107 (2.7) | 97 (2.4) | 0.523 |
| **Laboratory parameters, mean (SD)** |  |  |  |  |  |  |  |
| **Neutrophil, ×10^9^/L** | 5.66 (4.80) | 5.92 (4.01) | 0.001 |  | 5.91 (4.08) | 5.91 (3.99) | 0.939 |
| **Lymphocyte, ×10^9^/L** | 1.44 (13.37) | 1.35 (16.77) | 0.705 |  | 1.57 (18.75) | 1.36 (17.02) | 0.599 |
| **Glucose, mmol/L** | 7.28 (3.83) | 8.05 (4.16) | <0.001 |  | 7.97 (4.54) | 8.00 (4.11) | 0.743 |
| **High-density lipoprotein, mmol/L** | 1.13 (1.47) | 1.17 (1.94) | 0.189 |  | 1.16 (1.85) | 1.17 (1.97) | 0.875 |
| **Low-density lipoprotein, mmol/L** | 2.32 (1.61) | 2.33 (2.09) | 0.944 |  | 2.32 (1.63) | 2.33 (2.11) | 0.854 |
| **Alanine aminotransferase, IU/L** | 33.11 (83.95) | 34.32 (61.06) | 0.388 |  | 34.53 (86.57) | 34.24 (60.85) | 0.862 |
| **Aspartate aminotransferase, IU/L** | 41.56 (112.43) | 40.57 (75.78) | 0.592 |  | 40.85 (89.25) | 40.35 (76.52) | 0.789 |
| **Creatine, μmol/L** | 100.37 (195.45) | 92.63 (119.59) | 0.015 |  | 94.15 (103.13) | 92.87 (121.01) | 0.608 |
| **Glomerular filtration rate, ml/min** | 105.76 (144.20) | 103.65 (141.87) | 0.403 |  | 103.65 (140.49) | 103.65 (141.65) | 1 |
| **C-reactive protein, mg/L** | 49.82 (62.12) | 54.38 (64.22) | <0.001 |  | 53.38 (63.30) | 54.09 (64.06) | 0.615 |
| **Procalcitonin, ng/ml** | 1.88 (10.17) | 1.10 (6.35) | <0.001 |  | 1.29 (7.13) | 1.11 (6.43) | 0.235 |
| **Prothrombin time, s** | 14.67 (8.22) | 17.61 (11.05) | <0.001 |  | 16.75 (10.45) | 17.17 (10.79) | 0.079 |
| **Activated partial thromboplastin time, s** | 29.42 (11.74) | 26.09 (12.19) | <0.001 |  | 26.98 (11.03) | 26.48 (12.15) | 0.05 |
| **Cholesterol, mmol/L** | 4.25 (3.75) | 4.11 (3.08) | 0.031 |  | 4.16 (2.96) | 4.12 (3.12) | 0.486 |
| **Triglyceride, mmol/L** | 1.62 (4.67) | 1.54 (2.94) | 0.301 |  | 1.53 (4.01) | 1.55 (2.98) | 0.834 |
| **Alkaline phosphatase, IU/L** | 90.49 (72.25) | 81.97 (53.76) | <0.001 |  | 82.83 (44.71) | 82.23 (54.32) | 0.59 |
| **Gamma-glutamyl transpeptidase, IU/L** | 52.05 (90.46) | 54.50 (85.88) | 0.119 |  | 54.81 (89.71) | 54.28 (86.34) | 0.788 |
| **Albumin, g/L** | 38.30 (19.04) | 37.24 (32.15) | 0.007 |  | 37.63 (29.71) | 37.40 (32.61) | 0.74 |
| **Total bilirubin, μmol/L** | 14.11 (23.70) | 12.59 (12.18) | <0.001 |  | 12.65 (11.98) | 12.57 (12.29) | 0.751 |

^a^: Patients with severe disease in this study are defined as patients with severe and critical disease in the guidelines.
